# Prognostic differences in sepsis caused by gram-negative bacteria and gram-positive bacteria: a systematic review and meta-analysis

**DOI:** 10.1186/s13054-023-04750-w

**Published:** 2023-11-30

**Authors:** Aling Tang, Yi Shi, Qingqing Dong, Sihui Wang, Yao Ge, Chenyan Wang, Zhimin Gong, Weizhen Zhang, Wei Chen

**Affiliations:** 1 Longhua Hospital Affiliated to Shanghai University of Traditional Chinese Medicine, Shanghai, China; 2 Graduate School, Shanghai University of Traditional Chinese Medicine, Shanghai, China

**Keywords:** Sepsis, Gram-negative, Gram-positive, Bacteria, Prognosis

## Abstract

**Background:**

Bacteria are the main pathogens that cause sepsis. The pathogenic mechanisms of sepsis caused by gram-negative and gram-positive bacteria are completely different, and their prognostic differences in sepsis remain unclear.

**Methods:**

The PubMed, Web of Science, Cochrane Library, and Embase databases were searched for Chinese and English studies (January 2003 to September 2023). Observational studies involving gram-negative (G (−))/gram-positive (G (+)) bacterial infection and the prognosis of sepsis were included. The stability of the results was evaluated by sensitivity analysis. Funnel plots and Egger tests were used to check whether there was publication bias. A meta-regression analysis was conducted on the results with high heterogeneity to identify the source of heterogeneity. A total of 6949 articles were retrieved from the database, and 45 studies involving 5586 subjects were included after screening according to the Preferred Reporting Items for Systematic Reviews and Meta-Analyses guidelines. Twenty-seven high-quality studies and 18 moderate-quality studies were identified according to the Newcastle‒Ottawa Scale score. There was no significant difference in the survival rate of sepsis caused by G (−) bacteria and G (+) bacteria (OR 0.95, 95% CI 0.70–1.28). Subgroup analysis according to survival follow-up time showed no significant difference. The serum concentrations of C-reactive protein (CRP) (SMD = 0.39, 95% CI 0.02–0.76), procalcitonin (SMD = 1.95, 95% CI 1.32–2.59) and tumor necrosis factor-alpha (TNF-α) (MD = 0.31, 95% CI 0.25–0.38) in the G (−) bacterial infection group were significantly higher than those in the G (+) bacterial infection group, but there was no significant difference in IL-6 (SMD = 1.33, 95% CI − 0.18–2.84) and WBC count (MD = − 0.15, 95% CI − 0.96–00.66). There were no significant differences between G (−) and G (+) bacteria in D dimer level, activated partial thromboplastin time, thrombin time, international normalized ratio, platelet count, length of stay or length of ICU stay. Sensitivity analysis of the above results indicated that the results were stable.

**Conclusion:**

The incidence of severe sepsis and the concentrations of inflammatory factors (CRP, PCT, TNF-α) in sepsis caused by G (−) bacteria were higher than those caused by G (+) bacteria. The two groups had no significant difference in survival rate, coagulation function, or hospital stay. The study was registered with PROSPERO (registration number: CRD42023465051).

**Supplementary Information:**

The online version contains supplementary material available at 10.1186/s13054-023-04750-w.

## Introduction

Sepsis is a life-threatening organ dysfunction caused by a dysregulated host response to infection [1]. The host clears pathogens by activating the inflammatory response when pathogenic microorganisms invade the body. In sepsis, a systemic inflammatory response occurs due to the continuous activation of neutrophils and macrophages/monocytes, which leads to irreversible tissue damage and death [2]. The mechanism by which bacteria cause sepsis and septic shock involves bacterial components (cell wall, bacterial secretion products) and host responses (susceptibility, primary (immune) reaction, secondary (tissue) reaction, etc.) [3]. Initially, many studies suggested that the main microorganisms causing bacterial sepsis were gram-negative bacteria [4]. In the past 20 years, gram-positive bacteria, which are important pathogenic microorganisms that can also cause sepsis, have gradually attracted attention [5]. At present, the harmfulness of sepsis caused by G (−) bacteria and G (+) bacteria is still controversial. One study suggested that infection with G (+) bacteria caused a stronger host inflammatory response than infection with G (−) bacteria [6]. Another study suggested that there was no significant difference in the prognosis of sepsis caused by G (−) and G (+) bacteria [7]. To clarify the prognostic difference between sepsis caused by G (−) and G (+) bacteria, we conducted this systematic review and meta-analysis.

## Methods

This review followed the PRISMA Statement [8].

### Search strategy

The PubMed, Web of Science, Cochrane Library, and Embase databases were searched for Chinese and English studies in the past 20 years (January 2003 to September 2023). The complete search strategy is detailed in Additional file 1.

### Study selection

Two researchers performed the screening independently. The two researchers discussed with each other first if there were differences. A third investigator was consulted if disagreements could not be resolved. Screening was performed according to PRISMA guidelines.

### Inclusion and exclusion criteria

1. The following inclusion criteria were used: (1) human subjects; (2) clinical research; (3) observational studies; (4) patients with sepsis; and (5) studies including prognostic outcomes associated with G (−) and G (+) bacteria.
2. The following exclusion criteria were used: (1) in vitro studies and animal studies; (2) only infants (age < 3 years) included in the study; (3) intervention studies; (4) conference abstracts, comments, letters, case reports, and expert opinions; (5) the language was not Chinese or English; (6) duplicate articles; (7) incomplete data provision; (8) measurement data not provided or unable to be converted to mean and standard deviation; and (9) research data obtained from the database.

### Assessment of risk of bias

The risk of bias was assessed using the NOS by two researchers independently. The NOS consists of three parts: study population selection, comparability between groups, and outcome measures. The specific items and their scores are as follows: representativeness of the exposed cohort (1); selection of the nonexposed cohort (1); ascertainment of exposure (1); demonstration that the outcome of interest was not present at the start of the study (1); comparability of cohorts based on the design or analysis (2); assessment of outcome (1); sufficient follow-up length to allow outcomes to occur (1); and adequacy of follow-up of cohorts (1). Points are scored for each “yes” answer. According to the total score, studies were classified as high quality (7–9), moderate quality (4–6), and low quality (0–3).

### Data extraction

Two researchers extracted information from the included studies, including (1) basic research information: author, year of publication, country, study type, sample size, source of sample, and whether to be included in the meta-analysis, site of infection, underlying host disease, whether patients with immunodeficiency and chemoradiotherapy for malignant tumors were excluded, and treatment measures, whether subjects were enrolled only from the ICU; (2) primary outcome data: survival; and (3) secondary outcome data: inflammatory factor concentrations, Acute Physiology and Chronic Health Evaluation II (APACHE II) score, Sequential Organ Failure Assessment (SOFA) score, coagulation function, and length of hospital stay. It was preferred to obtain the relevant information directly from the publications. We obtained the data indirectly through the figures and datasets provided by the publication, if necessary.

### Statistical analysis

Data synthesis was performed using RevMan software 5.3 and Stata 12. We performed pooled analyses of survival across time points. We chose data for 28-day survival if data for multiple survival times were presented in the same study. For continuous variables, the standardized mean difference (SMD)/mean difference (MD) and 95% confidence interval of the two groups were calculated. The odds ratio (OR) between the two groups and the 95% confidence interval were calculated for binary variables. To test heterogeneity, *I*^2^ statistics were computed, and a *χ*2 test was performed. Heterogeneity was considered high when *I*^2^ > 50%, and a random-effects model was used. Heterogeneity was considered insignificant when *I*^2^ ≤ 50%, and a fixed-effects model was used. Subgroup analysis was performed for some of the results. Meta-regression analysis was used to obtain the source of heterogeneity for results with high heterogeneity and more than 10 included articles. Sensitivity analyses were used to assess the robustness of the results. Funnel plots and Egger’s test were used to detect publication bias. The significance for all two-sided *p* values was set at less than 0.05.

## Results

### Study selection and characteristics

**Fig. 1:**
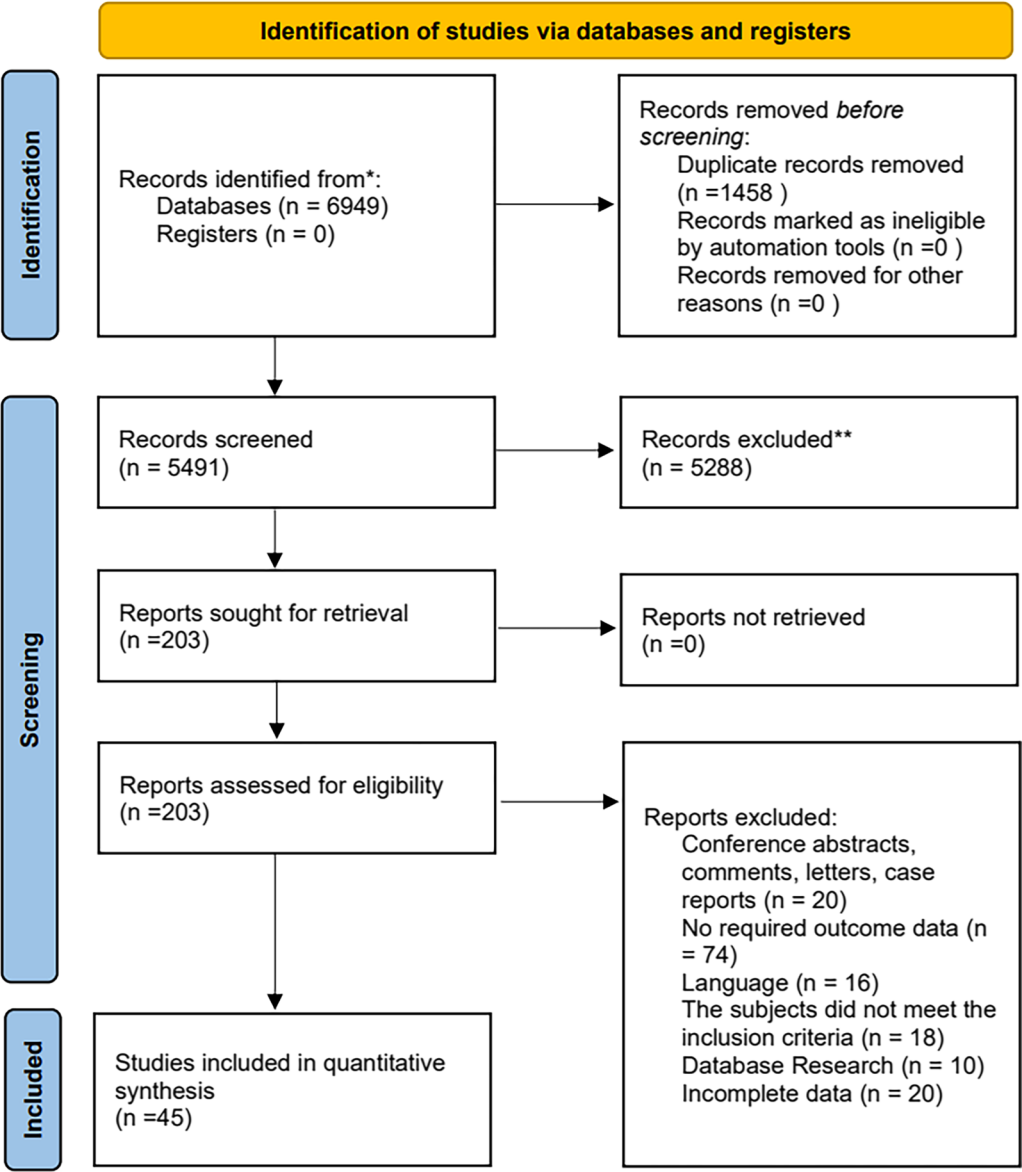
PRISMA flow diagram of study selection

A total of 6949 articles were initially retrieved from the database. After screening, a total of 45 studies were ultimately included (Fig. 1). All studies were conducted at a secondary or tertiary care center. The basic information of the included studies is shown in Table 1 and Additional file 2. According to the NOS score, the studies were divided into 27 high-quality studies and 18 medium-quality studies, and no low-quality studies were found. The scores are detailed in Additional file 3.

**Table 1:** Characteristics of all included studies

Author	Year	Country	Study design	*N*	Source of sample	Outcome	NOS score
Duan [9]	2023	China	Retrospective	121	Blood	1,5,6	8
Zhang [10]	2023	China	Prospective	107	Blood	1	8
Bilgin [11]	2023	Turkey	Retrospective	166	Blood	1,4	6
Chen [12]	2022	China	Prospective	152	Blood	2,5	6
Wu [13]	2022	China	Retrospective	74	Blood	5	8
Chen’ [14]	2022	China	Retrospective	104	Blood	1,4,5,6,	7
Huang [15]	2022	China	Prospective	46	Not mention	1,4,5,7	7
Liang [16]	2022	China	Retrospective	146	Blood	4	6
Hu [17]	2021	China	Prospective	35	Blood	5,3,6,7	6
Yan [18]	2021	China	Retrospective	221	Blood	1	8
Peng [19]	2020	China	Prospective	90	Blood	5,2,3	6
Leijte [20]	2020	France	Retrospective	141	Blood	1	8
Meng [21]	2019	China	Retrospective	69	Not mention	5,	6
Grande [22]	2019	Spain	Prospective	22	Blood	1,5	7
Gai [23]	2018	China	Retrospective	132	Blood	1,2,3,5,7	9
Zhang [24]	2018	China	Prospective	200	Blood	5	7
Liu [25]	2018	China	Retrospective	98	Blood	2,5	5
Lu [26]	2018	China	Prospective	26	Blood	4,5	7
Yunus [27]	2018	USA	Retrospective	188	Not mention	1,5,6	7
Lang [28]	2017	China	Prospective	50	Blood	2,5,6	6
Li [29]	2017	China	Prospective	82	Abdominal puncture	1,5	7
Liu [30]	2017	China	Prospective	120	Blood	4,5,	6
Gao [31]	2017	China	Retrospective	92	Blood	2,5,8	7
Liu’ [32]	2017	China	Retrospective	147	Blood	4,5	7
Tunjungputri [33]	2017	Netherlands	Prospective	32	Blood	5	7
Zhou [34]	2016	China	Prospective	112	Blood	5	6
Li [35]	2016	China	Retrospective	298	Blood	1,5	7
Surbatovic [36]	2015	Serbia	Prospective	145	Blood	1	6
Chen [37]	2015	China	Retrospective	136	Blood	5	7
Zhao [38]	2015	China	Retrospective	292	Blood	4,5	6
Guo [39]	2015	China	Retrospective	101	Blood	4	7
Aydemir [40]	2015	Turkey	Retrospective	192	Blood	1	6
Liu [41]	2014	China	Retrospective	126	Blood	2,5	6
Gao [42]	2014	China	Retrospective	73	Body fluids	5	7
Su [43]	2014	China	Prospective	26	Body fluids	5	6
Chen [44]	2014	China	Retrospective	132	Blood	5	6
Björnsson [45]	2014	Sweden	Prospective	22	Not mention	1	8
Nakajima [46]	2014	Japan	Prospective	14	Blood	1,5	6
Angeletti [47]	2013	Italy	Prospective	152	Blood	5	7
Labelle [48]	2012	USA	Retrospective	436	Blood	1,5	8
Abe [6]	2010	Japan	Retrospective	238	Blood	1,2,3,5,8	7
Cheng [49]	2007	China	Prospective	317	Body fluids	1	7
Feezor [50]	2003	USA	Prospective	52	Not mention	5	7
Blairon [51]	2003	Belgium	Prospective	35	Blood	5	6
Holub [52]	2003	Czech Republic	Prospective	20	Body fluids	1,2,3,5	7

1 Survival; 2 APACHE II; 3 SOFA score; 4 Septic shock/Severe sepsis; 5 Inflammatory biomarkers;6 Coagulation function; 7 Length of hospital stay; 8 ICU stay;

### Survival

**Fig. 2:**
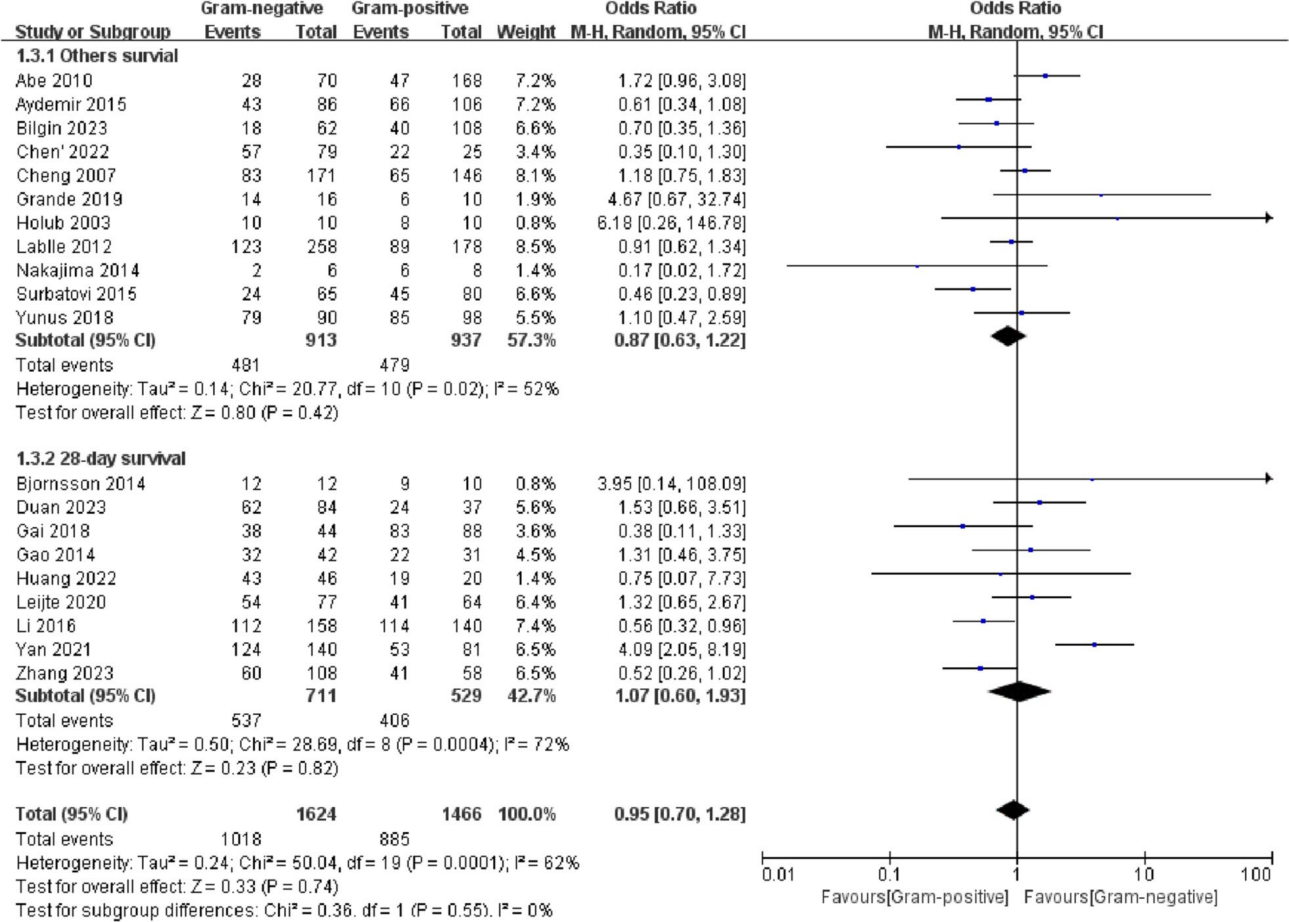
Forest plots of survival

A total of 20 studies had outcome measures associated with survival, including 28-day survival, hospital survival, ICU survival, and survival, without mention of follow-up time. A combined effect size analysis was performed for 20 studies (Fig. 2). We used a random-effects model due to the high heterogeneity of the results (*I*^2^ = 62%). The results showed that the survival rate of sepsis caused by G (+) bacteria (G (+) group) was not significantly different from that caused by G (−) bacteria (G (−) group) (OR 0.95, 95% CI 0.70–1.28, *p* = 0.74). No sources of heterogeneity were identified after a meta-regression analysis of 8 confounding factors (survival time points, sample size, whether subjects were enrolled only from the ICU, whether patients had only septic shock/severe sepsis, region, year of publication, whether only blood culture samples were collected, time of sampling, and the definition of sepsis) (Additional file 4). Subgroup analysis divided the studies into a 28-day survival group and an other survival group, and there was no difference between the two groups (*p* > 0.05). Egger’s test (*p* = 0.821) (Additional file 5) and funnel plot symmetry (Additional file 6) suggested that there was no significant publication bias (Fig. 2). Furthermore, we performed a subgroup analysis according to the definition of sepsis and found that the sepsis-1 group was less heterogeneous (*I*^2^ = 48%), suggesting that the definition of sepsis may be one of the sources of heterogeneity in this study (Additional file 7).

### Severity of sepsis

**Fig. 3:**
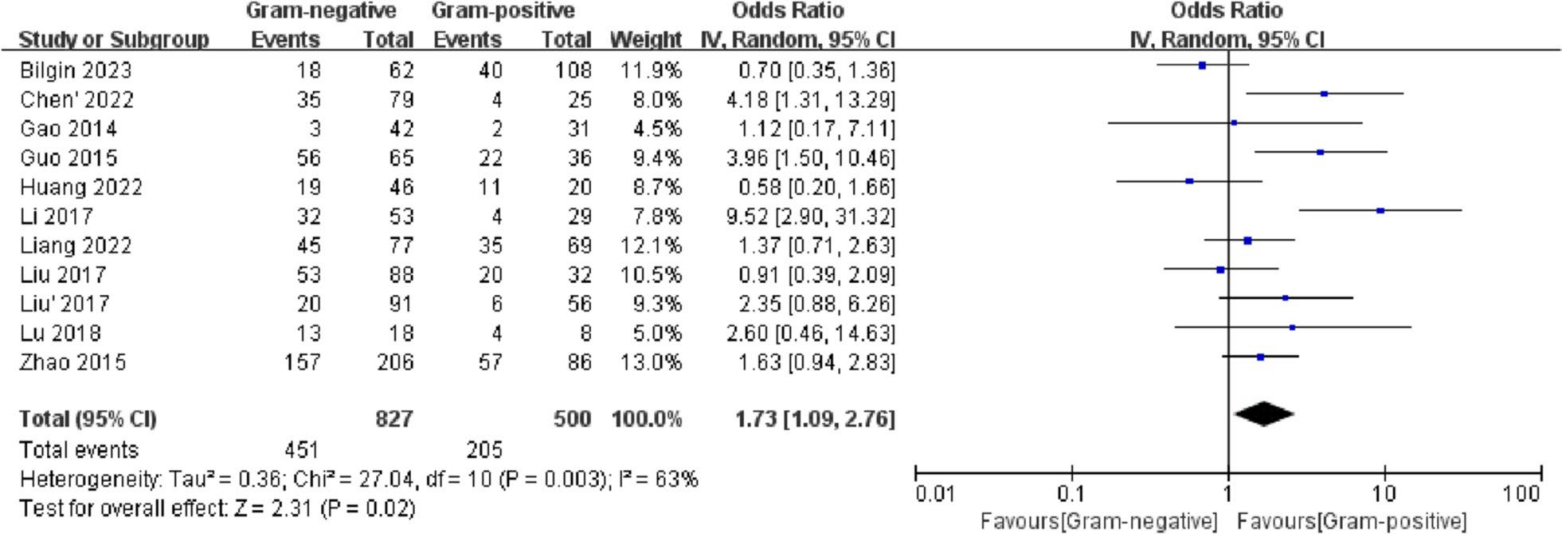
Forest plots of severe sepsis

Eleven studies reported the incidence of septic shock/severe sepsis. The random-effects model was used due to the high heterogeneity of the results (*I*^2^ = 63%). The incidence of septic shock/severe sepsis in the G (−) group was higher than that in the G (+) group (OR 1.73, 95% CI 1.09–2.76, *p* = 0.02). Meta-regression analysis suggested that whether patients were admitted only from the ICU might be the source of heterogeneity (*p* = 0.033) (Additional file 4). Egger’s test (*p* = 0.282) (Additional file 5) and funnel plot symmetry (Additional file 6) suggested that there was no significant publication bias (Fig. 3).

### APACHE II score

**Fig. 4:**
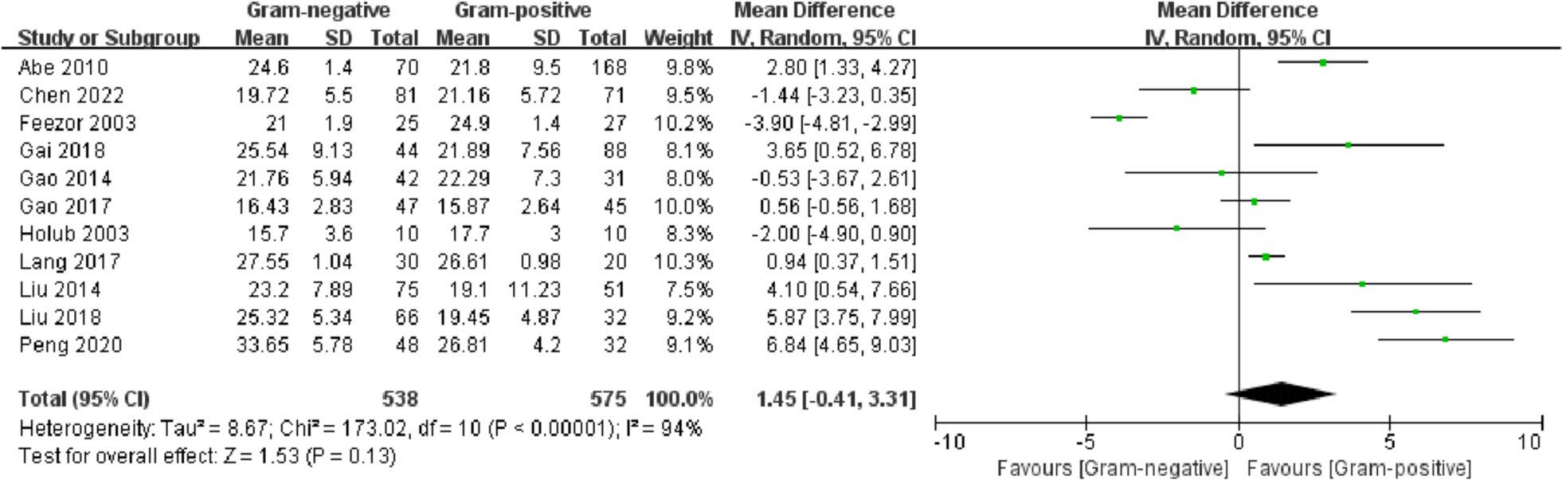
Forest plots of APACHE II score

A total of 10 studies reported APACHE II scores. The random-effects model was used due to the high heterogeneity of the results (*I*^2^ = 94%). The difference between the two groups was not significant (MD = 1.45, 95% CI − 0.41 ~ 3.31, *p* = 0.13). Meta-regression analysis revealed that the study region (*p* = 0.013 < 0.1), sample size (*p* = 0.041 < 0.1) and definition of sepsis (*p* = 0.093 < 0.1) may be sources of heterogeneity (Additional file 4). Egger’s test (*p* = 0.528) (Additional file 5) and funnel plot symmetry (Additional file 6) indicated that there was no significant publication bias (Fig. 4).

### SOFA score

**Fig. 5:**
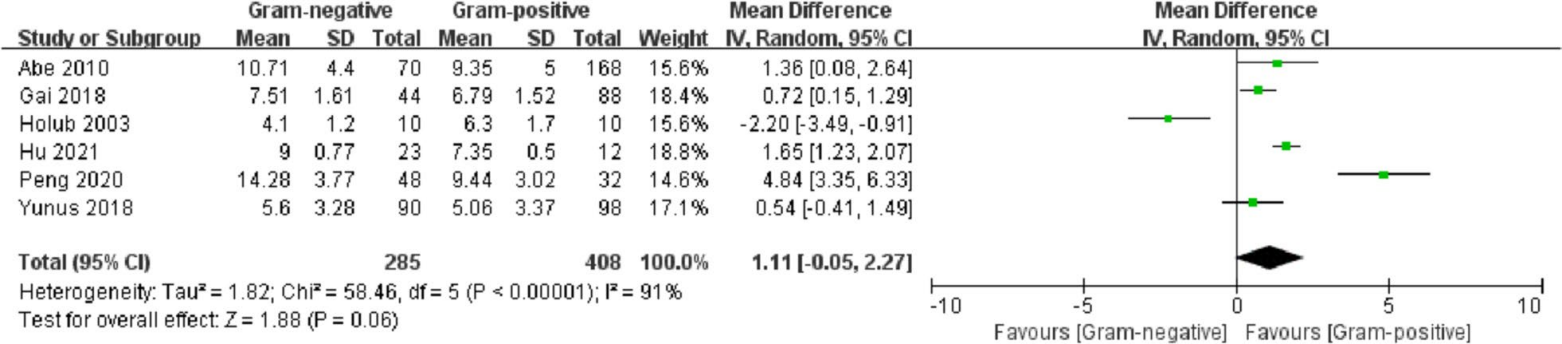
Forest plots of SOFA score

A total of five studies reported SOFA scores. There was no significant difference between the two groups (*p* = 0.06). After excluding a study [52] published 20 years ago, the SOFA score of the G (−) group was significantly higher than that of the G (+) group (MD = 1.66, 95% CI 0.69–2.64, *p* = 0.0008). Holub [52] was considered the source of heterogeneity (Fig. 5).

### Length of stay

**Fig. 6:**
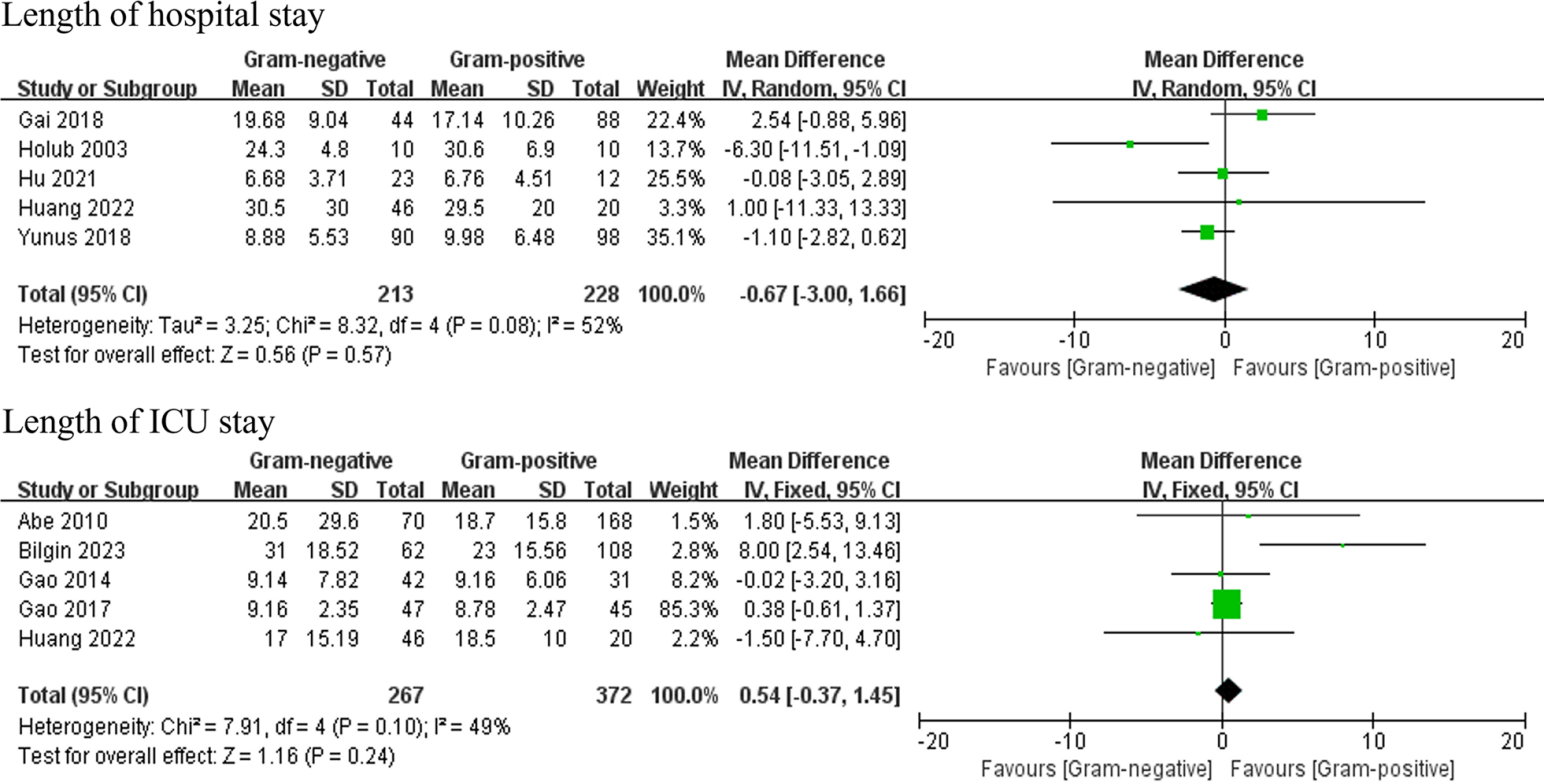
Forest plots of the length of stay

Four studies reported the length of hospital stay, and four studies reported the length of ICU stay. There was no significant difference between the G (−) group and the G (+) group (Fig. 6).

### WBCs

**Fig. 7:**
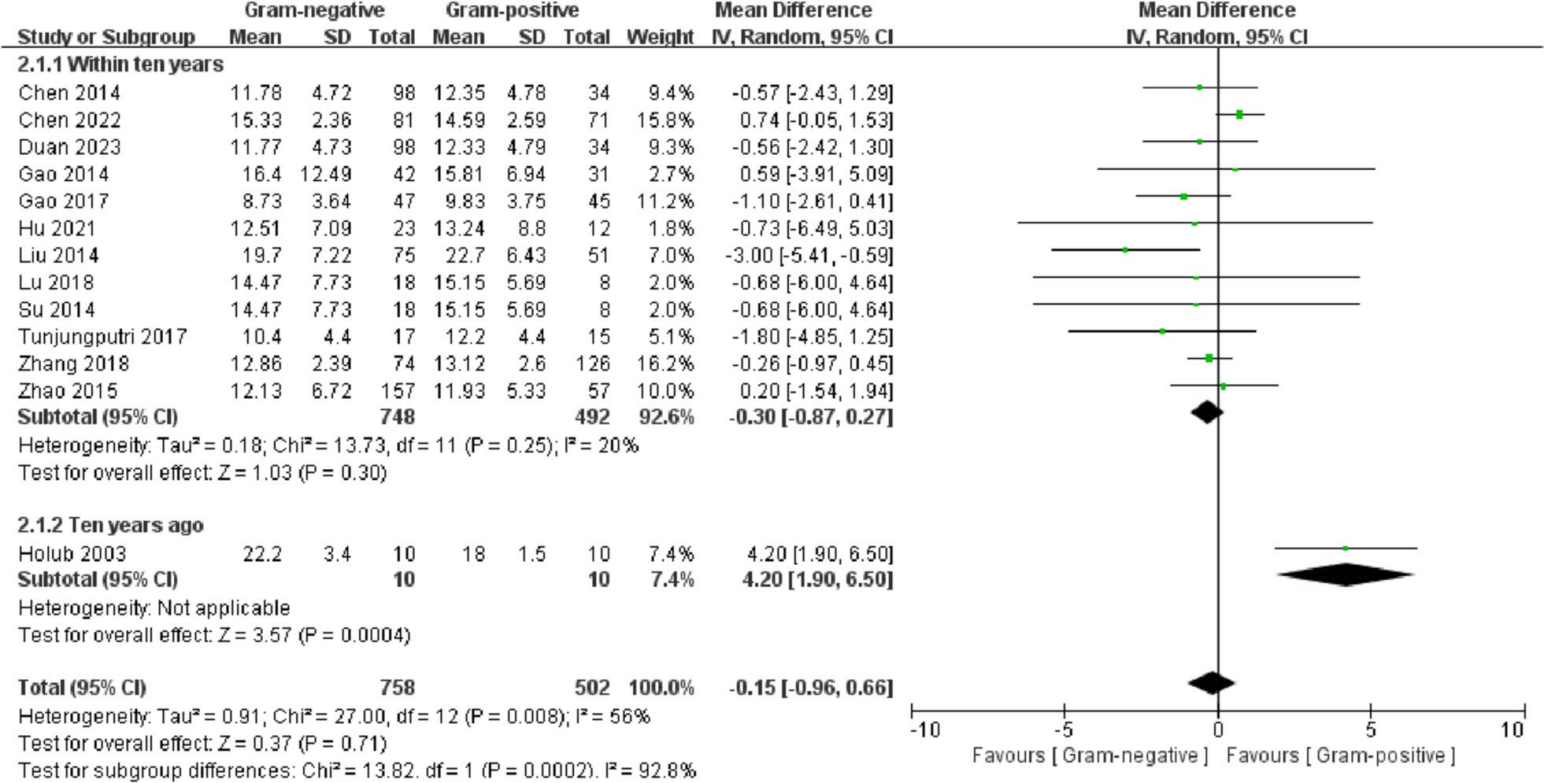
Forest plots of WBCs

A total of 12 studies mentioned white blood cells (WBCs). Subgroup analyses were performed according to the year of study publication. The combined effect sizes of studies published within ten years showed homogeneity (*I*^2^ = 20%). The random-effects model was used for analysis.The year of publication was considered a possible source of heterogeneity. The combined effect sizes of studies published within ten years showed that there was no significant difference between the G (−) and G (+) groups (MD = − 0.15, 95% CI − 0.96–00.66, *p* = 0.71) (Fig. 7).

### Inflammatory factors

#### CRP

**Fig. 8:**
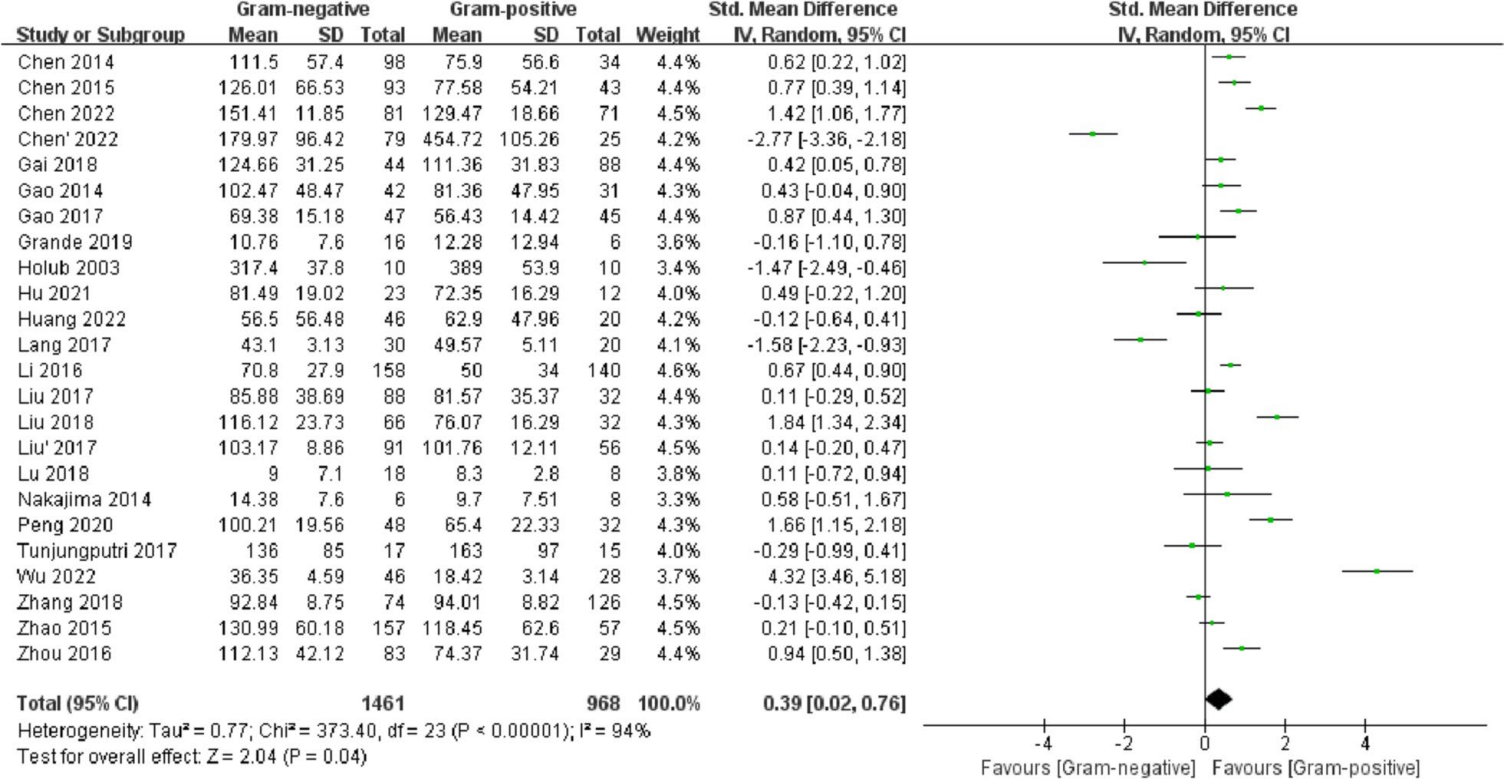
Forest plots of CRP concentration

CRP concentrations were reported in 23 studies. Heterogeneity among the studies was high (*I*^2^ = 94%), and a random-effects model was used. The serum CRP concentration of the G (−) group was higher than that of the G (+) group (SMD = 0.39, 95% CI 0.02–0.76, *p* = 0.04). Meta-regression analysis revealed that admission to the ICU (*p* = 0.055 < 0.1) and study region (*p* = 0.05 < 0.1) might be the sources of heterogeneity (Additional file 4). Egger’s test (*p* = 0.77) (Additional file 5) and funnel plot symmetry (Additional file 6) indicated that there was no significant publication bias (Fig. 8).

#### PCT

**Fig. 9:**
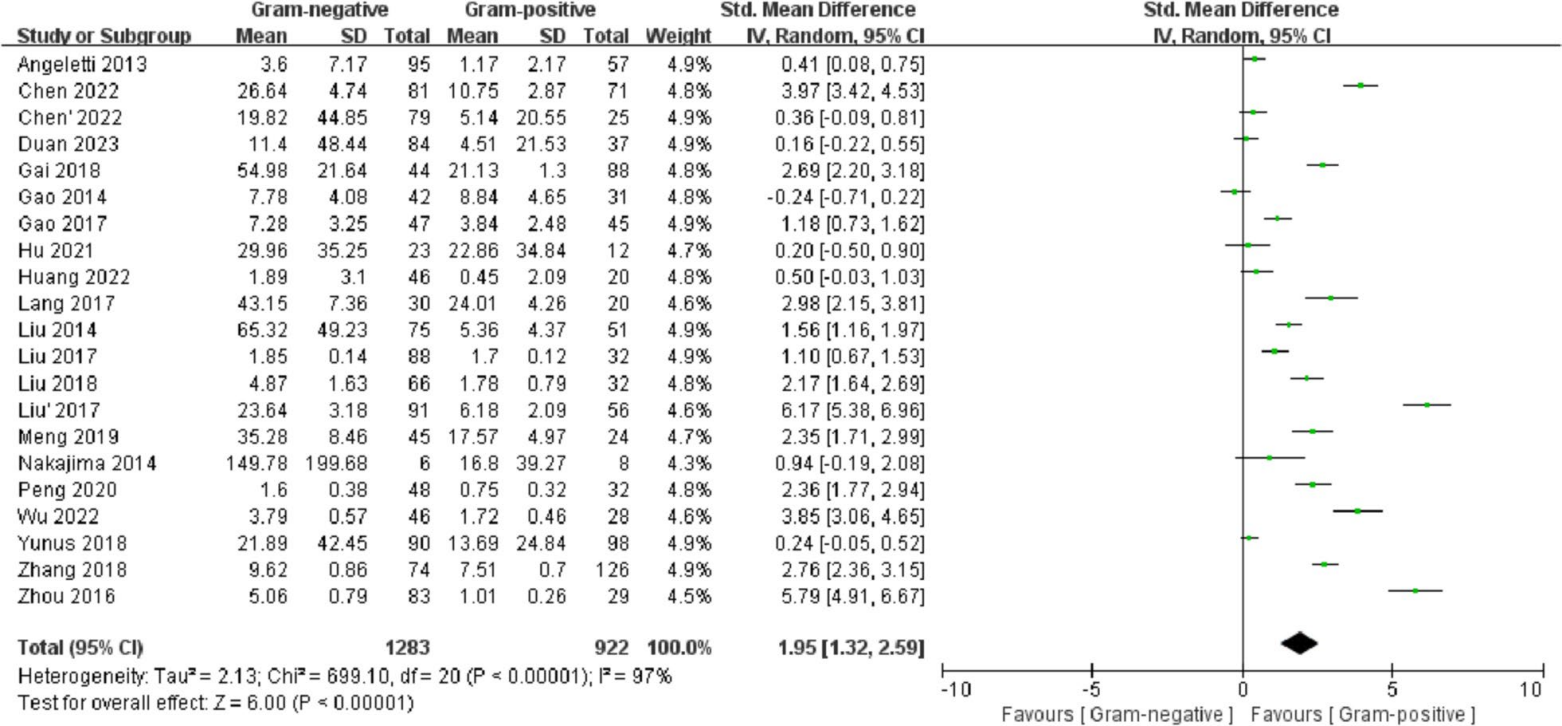
Forest plots of PCT concentration

Twenty studies reported serum PCT concentrations. A random-effects model was used due to the high interstudy heterogeneity (*I*^2^ = 97%). The serum PCT concentration of the G (−) group was significantly higher than that of the G (+) group (SMD = 1.95, 95% CI 1.32–2.59, *p* < 0.00001). Meta-regression analysis suggested that the study region (*p* = 0.061 < 0.1) might be the source of heterogeneity. Egger's test (*p* = 0.004) (Additional file 5) and funnel plot asymmetry (Additional file 6) showed publication bias (Fig. 9).

#### TNF-α and IL-6

Three studies reported serum concentrations of TNF-α, and five studies reported serum concentrations of interleukin-6 (IL-6). The serum TNF-α concentration in the G (−) group was significantly higher than that in the G (+) group (MD = 0.31, 95% CI 0.25–0.38, *p* < 0.00001). There was no significant difference in serum IL-6 concentration between the two groups (SMD = 1.33, 95% CI − 0.18–2.84, *p* = 0.08) (Additional file 7).

### Coagulation function

Five studies reported D-D concentration, 2 studies reported APTT, 2 studies reported TT, 2 studies reported INR and 4 studies reported platelet (PLT) counts. After combining effect values, it was found that the G (−) and G (+) groups were not significantly different (Additional file 7).

#### Sensitivity analysis

Sensitivity analyses were performed separately for all results, which indicated that each result was stable.

## Discussion

The meta-analysis revealed that sepsis caused by G (−) bacteria was more severe than that caused by G (+) bacteria. In addition, the concentrations of inflammatory factors in the G (−) group were significantly higher than those in the G (+) group. However, our study found that there was no significant difference in survival rate, coagulation function, length of stay, APACHE II score, or SOFA score between the G (−) and G (+) groups. We identified some sources of heterogeneity by meta-regression analysis, subgroup analysis, funnel plot, and Egger's test. Sensitivity analyses suggested that all results were stable.

Bacteria are one of the most common pathogens that cause sepsis, and there are significant differences in pathogenic mechanisms between G (−) bacteria and G (+) bacteria [4]. There are fundamental differences in the host response to infection with G (−) and G (+) bacteria, which are related to differences in their composition and structure [53].

Bacterial cell wall components include lipopolysaccharide (LPS), peptidoglycan (PGN), and lipoteichoic acid (LTA). LPS is the main component of the G (−) outer membrane. LPS and other cell wall components are released when bacteria multiply or die in the host. The toxic fraction lipid A causes the body's immune response [54]. The structure of the G (+) cell wall is different from that of G (−) bacteria, and its cell membrane is a single-cell membrane with PGN, LTA, etc., as the main components [55]. An experimental study found a significant increase in plasma concentrations of TNF-α, IFN-γ, and IL-10 one hour after intraperitoneal injection of LPS, whereas no significant increase was found after intraperitoneal injection of LTA [56]. In our meta-analysis, the serum concentrations of multiple proinflammatory factors were also elevated in patients with sepsis caused by G (−) bacteria. The results suggest that G (−) bacterial infection may cause a more severe systemic inflammatory response, which may be one of the important reasons for the increased severity of sepsis.

A total of 5259 patients had at least one positive microbiological culture in a study involving 15,202 subjects. Sixty-seven percent were gram-negative bacteria, 37% were gram-positive bacteria, and 16% were fungi [57]. The main site of infection was the lung (44.8%), followed by the abdomen (31.5%), urinary tract (6.2%), central venous catheter (4.6%), soft tissue (3.1%), and surgical wound (3.1%) [58]. *Staphylococcus aureus* and Pseudomonas species were the most common G (−) bacteria and G (+) bacteria. Different microorganisms and sites of infection interact in determining mortality [59]. As a reference, we recorded the information about the site of infection from each study. In general, many studies have suggested that sepsis caused by G (−) bacteria is more severe than that caused by G (+) bacteria [6]. A growing number of studies have different points of view. The pathogens causing sepsis used to be mainly G (−) bacteria, but they are being gradually replaced by G (+) bacteria [60]. The incidence and mortality of sepsis caused by gram-positive bacteria are increasing, which may be related to the resistance of G (+) bacteria [61]. The development of antibacterial drugs is underway, but the harmfulness of G (+) bacteria is not matched by the attention it receives [60]. In this study, G (−) bacteria caused more severe sepsis, but there were no differences in survival or length of hospital stay between the G (−) and G (+) groups. Bacteremia is thought to be associated with poor prognosis in sepsis. We performed a subgroup analysis of whether the patients were complicated with bacteremia and found that there was no difference in survival between the two groups. It is important to increase awareness of sepsis caused by G (+) bacteria.

Polymicrobial infection is a scenario that should be considered. Studies have shown that polymicrobial infection is a risk factor for severe sepsis [62]. The mortality of G (+) and G (−) infected patients was significantly increased when they were coinfected with COVID-19 [63]. The proportion of sepsis infections caused by fungi is increasing [64]. It is uncertain whether coinfection with other microorganisms is responsible for the difference in prognosis between the G (−) and G (+) groups. Some studies included only subjects with a single positive culture, while others included subjects with polymicrobial infection. This may have influenced our results.

Our study has some limitations. 1) Studies in languages other than Chinese or English were excluded, which may have resulted in an incomplete number of included studies. 2) Some results of this meta-analysis showed high heterogeneity. We identified some sources of heterogeneity through a series of methods, but some sources of heterogeneity are still unclear. 3) Because there are significant differences between children and adults in the prognosis and physiology of sepsis, we excluded studies involving only infants. However, we did not perform separate analyses for the other age groups. 4) Polymicrobial infection was not considered as a variable in this study.

## Conclusion

In conclusion, sepsis caused by G (−) bacteria has higher serum inflammatory factor concentrations and greater disease severity than sepsis caused by G (+) bacteria. However, there was no significant difference in survival rate, length of stay, APACHE II score, SOFA score, or coagulation function between the two groups. This provides suggestions for the treatment of sepsis. The pathophysiological differences between G (−) and G (+) bacteria causing sepsis still need to be further studied.

### Supplementary Information

**Additional file 1**. Search strategy.

**Additional file 2**. Characteristics of included studies.

**Additional file 3**. NOS Score.

**Additional file 4**. Meta-regression.

**Additional file 5**. Egger's test.

**Additional file 6**. Plot of funnel.

**Additional file 7**. Forest plots.

## Data Availability

All data generated or analyzed during this study is included in this published article [and its supplementary information files.

## Abbreviations

G (−): Gram-negative
G (+): Gram-positive
PRISMA: Preferred Reporting Items for Systematic Reviews and Meta-Analyses
NOS: Newcastle–Ottawa scale
APACHE II: Acute physiology and chronic health evaluation
SOFA: Sequential organ failure assessment
SMD: Standardized mean difference
MD: Mean difference
OR: Odds ratio
CI: Confidence interval
CRP: Serum C-reactive protein
PCT: Procalcitonin
TNF-α: Tumor necrosis factor-alpha
IL-6: Interleukin-6
WBC: White blood cells
D-D: D dimer
APTT: Activated partial thromboplastin time
TT: Thrombin time
INR: International normalized ratio
PLT: Platelet
LPS: Lipopolysaccharides
LTA: Lipoteichoic acid
PGN: Peptidoglycan
TLR4: Toll-like receptor 4
TRP: Transient receptor potential
