# Supplementary material for: Prognostic differences in sepsis caused by gram-negative bacteria and gram-positive bacteria: a systematic review and meta-analysis
Source: Crit Care. 2023 Nov 30;27:467. doi: 10.1186/s13054-023-04750-w (PMC10691150; doi:10.1186/s13054-023-04750-w)
Supplement: Supplementary file 1 — Additional file 1. Search strategy. [file 13054_2023_4750_MOESM1_ESM.docx]

|  | **Search terms** |
| --- | --- |
|  | ***PubMed*** |
| **#4** | #1 AND #2 AND #3 |
| **#3** | ((Gram-negative [Title/Abstract]) OR (Gram-Negative Bacterial Infections [Title/Abstract])) OR (Gram-Negative Bacteria [Title/Abstract]) |
| **#2** | ((Gram-positive [Title/Abstract]) OR (Gram-Positive Bacterial Infections [Title/Abstract])) OR (Gram-Positive Bacteria [Title/Abstract]) |
| **#1** | (((((sepsis [Title/Abstract]) OR (septic [Title/Abstract])) OR (Septic shock [Title/Abstract])) OR (Severe sepsis [Title/Abstract]))) |
|  | ***Web of Science*** |
| **#4** | #1 AND #2 AND #3 |
| **#3** | TS= (Gram-negative OR Gram-Negative Bacterial Infections OR Gram-Negative Bacteria) |
| **#2** | TS= (Gram-positive OR Gram-Positive Bacterial Infections OR Gram-Positive Bacteria) |
| **#1** | TS= (sepsis OR septic OR Septic shock OR Severe sepsis) |
|  | ***Cochrane Library*** |
| **#4** | #1 and #2 AND #3 |
| **#3** | Gram-positive:kw,ti,ab or Gram-Positive Bacterial Infections:kw,ti,ab or Gram-Positive Bacteria:kw,ti,ab |
| **#2** | Gram-negative:kw,ti,ab or Gram-Negative Bacterial Infections:kw,ti,ab or Gram-Negative Bacteria:kw,ti,ab |
| **#1** | sepsis:kw,ti,ab or septic:kw,ti,ab or Septic shock:kw,ti,ab or Severe sepsis:kw,ti,ab |
|  | ***EMBASE*** |
| **#4** | #1 and #2 AND #3 |
| **#3** | Gram-positive:kw,ti,ab or Gram-Positive Bacterial Infections:kw,ti,ab or Gram-Positive Bacteria:kw,ti,ab |
| **#2** | Gram-negative:kw,ti,ab or Gram-Negative Bacterial Infections:kw,ti,ab or Gram-Negative Bacteria:kw,ti,ab |
| **#1** | sepsis:kw,ti,ab or septic:kw,ti,ab or Septic shock:kw,ti,ab or Severe sepsis:kw,ti,ab |
|  | ***China National Knowledge Infrastructure*** |
| **#3** | #1 and #2 |
| **#2** | TS= (Vascular Endothelial Growth Factor OR VEGF* OR Vascular Endothelial Growth Factor A OR Vascular Endothelial Growth Factor B OR VEGFA protein, human OR Vascular Endothelial Growth Factor Receptor-2 OR Vascular Endothelial Growth Factor Receptor-1 OR Receptors, Vascular Endothelial Growth Factor OR VEGFB protein, human OR Vascular Endothelial Growth Factor D OR Vascular Endothelial Growth Factor C) |
| **#1** | TS= (sepsis OR septic OR Septic shock OR Severe sepsis) |
