# Supplementary material for: Prognostic differences in sepsis caused by gram-negative bacteria and gram-positive bacteria: a systematic review and meta-analysis
Source: Crit Care. 2023 Nov 30;27:467. doi: 10.1186/s13054-023-04750-w (PMC10691150; doi:10.1186/s13054-023-04750-w)
Supplement: Supplementary file 2 — Additional file 2. Characteristics of included studies. [file 13054_2023_4750_MOESM2_ESM.docx]

**Characteristics of included studies**

| **Author** | **Site of infection(n) ^1^** | **Underlying host illnesses ^2^** | **Host immune status ^3^** | **Treatment** | **Definition of sepsis** | **ICU treatment** |
| --- | --- | --- | --- | --- | --- | --- |
| Duan ^[10]^ | Not mention | Not mention | Not mention | Not mention | Sepsis 3 | Yes |
| Zhang ^[11]^ | Not mention | Yes | Yes | Not mention | Sepsis 3 | No |
| Bilgin ^[12]^ | Not mention | Not mention | Not mention | Not mention | Sepsis 3 | Yes |
| Chen ^[13]^ | Not mention | Not mention | Yes | Symptomatic treatment + Standardized anti-infective treatment | Sepsis 3 | No |
| Wu ^[14]^ | a(39),f(18),g(12),h(5) | Not mention | Yes | Not mention | Sepsis 2 | No |
| Chen’ ^[15]^ | a(29),f(83),h(27),i(16) | Yes | Yes | Not mention | Sepsis 3 | No |
| Huang ^[16]^ | 1(27),2(17),3(9),4(8),5(5) | Yes | Yes | Not mention | Sepsis 3 | Yes |
| Liang ^[17]^ | Not mention | Yes | Yes | Not mention | Sepsis 3 | Yes |
| Hu ^[18]^ | Not mention | Not mention | No | Not mention | Sepsis 3 | Yes |
| Yan ^[19]^ | g(221) | Not mention | No | Not mention | Sepsis 3 | No |
| Peng ^[20]^ | a(32),e(14),g(14),h(9),j(11) | Not mention | Yes | Not mention | Sepsis 2 | No |
| Leijte ^[21]^ | a(44),g(107),h(29) | Not mention | Yes | Accord-  ing to the Surviving Sepsis Campaign | Sepsis 3 | Yes |
| Meng ^[22]^ | a(32),b(10),g(50),,h(11),i(10) | Not mention | No | Not mention | Sepsis 3 | Yes |
| Grande ^[23]^ | Not mention | Not mention | No | Not mention | Sepsis 3 | No |
| Gai ^[24]^ | Not mention | Not mention | No | Not mention | Sepsis 1 | No |
| Zhang ^[25]^ | Not mention | Not mention | No | Not mention | Sepsis 2 | No |
| Liu ^[26]^ | Not mention | Not mention | Yes | Not mention | Sepsis 2 | Yes |
| Lu ^[27]^ | a, f, h, i, j | Yes | Yes | Not mention | Sepsis 1 | No |
| Yunus ^[28]^ | a(166),h(71),e(56),l(44) | Yes | No | Not mention | Sepsis 3 | No |
| Lang ^[29]^ | Not mention | Yes | No | Not mention | Sepsis 2 | Yes |
| Li ^[30]^ | g(82) | Not mention | No | Symptomatic treatment | Sepsis 1 | No |
| Liu ^[31]^ | a(61),d(16),e(17),g(33),h(20) | Not mention | No | Not mention | Sepsis 1 | No |
| Gao ^[32]^ | Not mention | Not mention | No | Not mention | Sepsis 1 | No |
| Liu’ ^[33]^ | Not mention | Not mention | Yes | Not mention | Sepsis 2 | Yes |
| Tunjungputri^[34]^ | a(2),d(5),e(9),h(11),j(2),m(2),n(1) | Not mention | Only malignant tumors were excluded | Not mention | Not mention | No |
| Zhou ^[35]^ | Not mention | Not mention | Yes | Not mention | Sepsis 2 | Yes |
| Li ^[36]^ | a(148),d(26),e(33),f(52),h(36), | Not mention | No | Not mention | Sepsis 2 | No |
| Surbatovic ^[37]^ | g(145) | Not mention | No | Accord-  ing to the Surviving Sepsis Campaign | Sepsis 2 | Yes |
| Chen ^[38]^ | a(86),g(23),h(28),j(15) | China | Yes | Standard sepsis treatment | Sepsis 2 | No |
| Zhao ^[39]^ | Not mention | Not mention | Immunodeficiency, lung cancer, and thyroid cancer were excluded | Not mention | Sepsis 2 | Yes |
| Guo ^[40]^ | Not mention | Not mention | No | Not mention | Not mention | Yes |
| Aydemir ^[41]^ | a(50),e(14),g(30),h(29),m(69) | Not mention excluded | Only malignant tumors were excluded | Not mention | Sepsis 1 | Yes |
| Liu ^[42]^ | Not mention | Not mention | No | Not mention | Not mention | Yes |
| Gao ^[43]^ | a, b, d, h, f, m | Yes | No | Not mention | Sepsis 2 | Yes |
| Su ^[44]^ | a(43),e(4),f(5),h(7),i(2),o(1) | Not mention | Yes | Not mention | Sepsis 2 | No |
| Chen ^[45]^ | Not mention | Not mention | Yes | Not mention | Sepsis 2 | Yes |
| Björnsson ^[46]^ | Not mention | Yes | No | According to Malmö University Hospital’s guidelines | Sepsis 1 | No |
| Nakajima ^[47]^ | Not mention | Yes | No | Not mention | Sepsis 1 | No |
| Angeletti ^[48]^ | a(14),d(11),e(36),g(57),(42),k(8),i(4),m(28), | Yes | No | Not mention | Sepsis 1 | No |
| Labelle ^[49]^ | a(156),d(14),e(35),g(54),h(65),i(2),k(11),l(3),m(96), | Not mention | Not mention | Symptomatic treatment + Standardized anti-infective treatment | Sepsis 1 | No |
| Abe ^[50]^ | Not mention | Not mention | Yes | Not mention | Sepsis 1 | Yes |
| Cheng ^[51]^ | a(168),b(90),d(93),e(188),g(230), h(23) | Yes | No | Not mention | Sepsis 1 | Yes |
| Feezor ^[52]^ | Not mention | Not mention | Immunodeficiency was excluded | Not mention | Sepsis 1 | No |
| Blairon ^[53]^ | a(15),b(5),e(9),g(8) | Not mention | Not mention | Not mention | Sepsis 1 | Yes |
| Holub ^[54]^ | a(3),c(1),e(3),i(23),k(1) | Not mention | Yes | Not mention | Sepsis 1 | No |

^1^ a Respiratory infection, b Bloodstream infection, c Thoracic or abdominal infection, d Wound or soft tissue infection, e Other infections, f Digestive System infections, g Abdominal infection, h Urinary tract infections, i Infection of the central nervous system, j Biliary tract infection, k Heart-related infections, l Musculoskeletal Infections, m Intravascular catheter-related infection, n Endometrial Infections, o Liver abscess.

^2^ Whether the included subjects had other underlying diseases or not.

^3^ Whether subjects with immunodeficiency or a history of radiotherapy and chemotherapy for cancer were excluded.
