# Supplementary material for: Prognostic differences in sepsis caused by gram-negative bacteria and gram-positive bacteria: a systematic review and meta-analysis
Source: Crit Care. 2023 Nov 30;27:467. doi: 10.1186/s13054-023-04750-w (PMC10691150; doi:10.1186/s13054-023-04750-w)
Supplement: Supplementary file 3 — Additional file 3. NOS Score. [file 13054_2023_4750_MOESM3_ESM.docx]

**NOS Score**

| Study | Selection | | | | Comparability of Cohorts on the Basis of the Design or Analysis | OUTCOME | | | Scores |
| --- | --- | --- | --- | --- | --- | --- | --- | --- | --- |
|  | Representativeness of the Exposed Cohort | Selection of the Non-Exposed Cohort | Ascertainment of Exposure | Demonstration That Outcome of Interest Was Not Present at Start of Study |  | Assessment of Outcome | Was Follow-Up Long Enough for Outcomes to Occur | Adequacy of Follow Up of Cohorts |  |
| Duan  2023 | ★ | ★ | ★ |  | ★★ | ★ | ★ | ★ | 8 |
| Zhang 2023 | ★ | ★ | ★ |  | ★★ | ★ | ★ | ★ | 8 |
| Bilgin 2023 |  | ★ | ★ |  | ★★ | ★ | - | ★ | 6 |
| Chen 2022 | ★ | ★ | ★ |  | ★★ | ★ |  |  | 6 |
| Wu 2022 | ★ | ★ | ★ |  | ★★ | ★ | ★ | ★ | 8 |
| Chen 2022 | ★ | ★ | ★ |  | ★★ | ★ |  | ★ | 7 |
| Huang  2022 |  | ★ | ★ |  | ★★ | ★ | ★ | ★ | 7 |
| Liang 2022 |  | ★ | ★ |  | ★★ | ★ |  | ★ | 6 |
| Hu  2021 |  | ★ | ★ |  | ★★ | ★ |  | ★ | 6 |
| Yan 2021 | ★ | ★ | ★ |  | ★★ | ★ | ★ | ★ | 8 |
| Peng 2020 | ★ | ★ | ★ |  | ★★ | ★ |  | ★ | 6 |
| Leijte 2020 |  | ★ | ★ | ★ | ★★ | ★ | ★ | ★ | 8 |
| Meng  2019 |  | ★ | ★ |  | ★★ | ★ |  | ★ | 6 |
| Grande 2019 | ★ | ★ | ★ |  | ★★ | ★ |  | ★ | 7 |
| Gai 2018 | ★ | ★ | ★ | ★ | ★★ | ★ | ★ | ★ | 9 |
| Zhang  2018 | ★ | ★ | ★ |  | ★★ | ★ |  | ★ | 7 |
| Liu  2018 |  | ★ | ★ |  | ★★ | ★ |  |  | 5 |
| Lu  2018 | ★ | ★ | ★ |  | ★★ | ★ |  | ★ | 7 |
| Yunus  2018 | ★ | ★ | ★ |  | ★★ | ★ |  | ★ | 7 |
| Lang  2017 |  | ★ | ★ |  | ★★ | ★ |  | ★ | 6 |
| Li  2017 | ★ | ★ | ★ |  | ★★ | ★ |  | ★ | 7 |
| Liu  2017 |  | ★ | ★ |  | ★★ | ★ |  | ★ | 6 |
| Gao  2017 | ★ | ★ | ★ |  | ★★ | ★ |  | ★ | 7 |
| Liu  2017 | ★ | ★ | ★ |  | ★★ | ★ |  | ★ | 7 |
| Tunjungputri  2017 | ★ | ★ | ★ |  | ★★ | ★ |  | ★ | 7 |
| Zhou  2016 |  | ★ | ★ |  | ★★ | ★ |  | ★ | 6 |
| Li  2016 | ★ | ★ | ★ |  | ★★ | ★ |  | ★ | 7 |
| Surbatovic  2015 |  | ★ | ★ |  | ★★ | ★ |  | ★ | 6 |
| Chen  2015 | ★ | ★ | ★ |  | ★★ | ★ |  | ★ | 7 |
| Zhao  2015 |  | ★ | ★ |  | ★★ | ★ |  | ★ | 6 |
| Guo  2015 | ★ | ★ | ★ |  | ★★ | ★ |  | ★ | 7 |
| Aydemir 2015 |  | ★ | ★ |  | ★★ | ★ |  | ★ | 6 |
| Liu  2014 |  | ★ | ★ |  | ★★ | ★ |  | ★ | 6 |
| Gao  2014 |  | ★ | ★ |  | ★★ | ★ | ★ | ★ | 7 |
| Su  2014 |  | ★ | ★ |  | ★★ | ★ |  | ★ | 6 |
| Diao  2014 |  | ★ | ★ |  | ★★ | ★ |  | ★ | 6 |
| Björnsson  2014 | ★ | ★ | ★ |  | ★★ | ★ | ★ | ★ | 8 |
| Nakajima  2014 |  | ★ | ★ |  | ★★ | ★ |  | ★ | 6 |
| Angeletti 2013 | ★ | ★ | ★ |  | ★★ | ★ |  | ★ | 7 |
| Labelle 2012 | ★ | ★ | ★ | ★ | ★★ | ★ |  | ★ | 8 |
| Abe  2010 | ★ | ★ | ★ |  | ★★ | ★ |  | ★ | 7 |
| Cheng 2007 |  | ★ | ★ |  | ★★ | ★ | ★ | ★ | 7 |
| Feezor 2003 | ★ | ★ | ★ |  | ★★ | ★ |  | ★ | 7 |
| Blairon 2003 |  | ★ | ★ |  | ★★ | ★ |  | ★ | 6 |
| Holub 2003 | ★ | ★ | ★ |  | ★★ | ★ |  | ★ | 7 |
