# Supplementary material for: Prognostic differences in sepsis caused by gram-negative bacteria and gram-positive bacteria: a systematic review and meta-analysis
Source: Crit Care. 2023 Nov 30;27:467. doi: 10.1186/s13054-023-04750-w (PMC10691150; doi:10.1186/s13054-023-04750-w)
Supplement: Supplementary file 4 — Additional file 4. Meta-regression. [file 13054_2023_4750_MOESM4_ESM.docx]

Meta-regression

- Survival (survival time points, sample size, whether subjects were enrolled only from the ICU, whether patients had only septic shock/severe sepsis, region, year of publication, whether only blood culture samples were collected, time of sampling, and the definition of sepsis).
- Severe sepsis (sample size, whether subjects were enrolled only from the ICU, year of publication, whether only blood culture samples were collected, time of sampling, and the definition of sepsis).
- CRP (sample size, whether subjects were enrolled only from the ICU, whether patients had only septic shock/severe sepsis, region, year of publication, whether only blood culture samples were collected, time of sampling, and the definition of sepsis).
- PCT (sample size, whether subjects were enrolled only from the ICU, region, year of publication, whether only blood culture samples were collected, time of sampling, and the definition of sepsis).
- APACHE Ⅱ (sample size, whether subjects were enrolled only from the ICU, region, year of publication, whether only blood culture samples were collected, time of sampling, and the definition of sepsis).


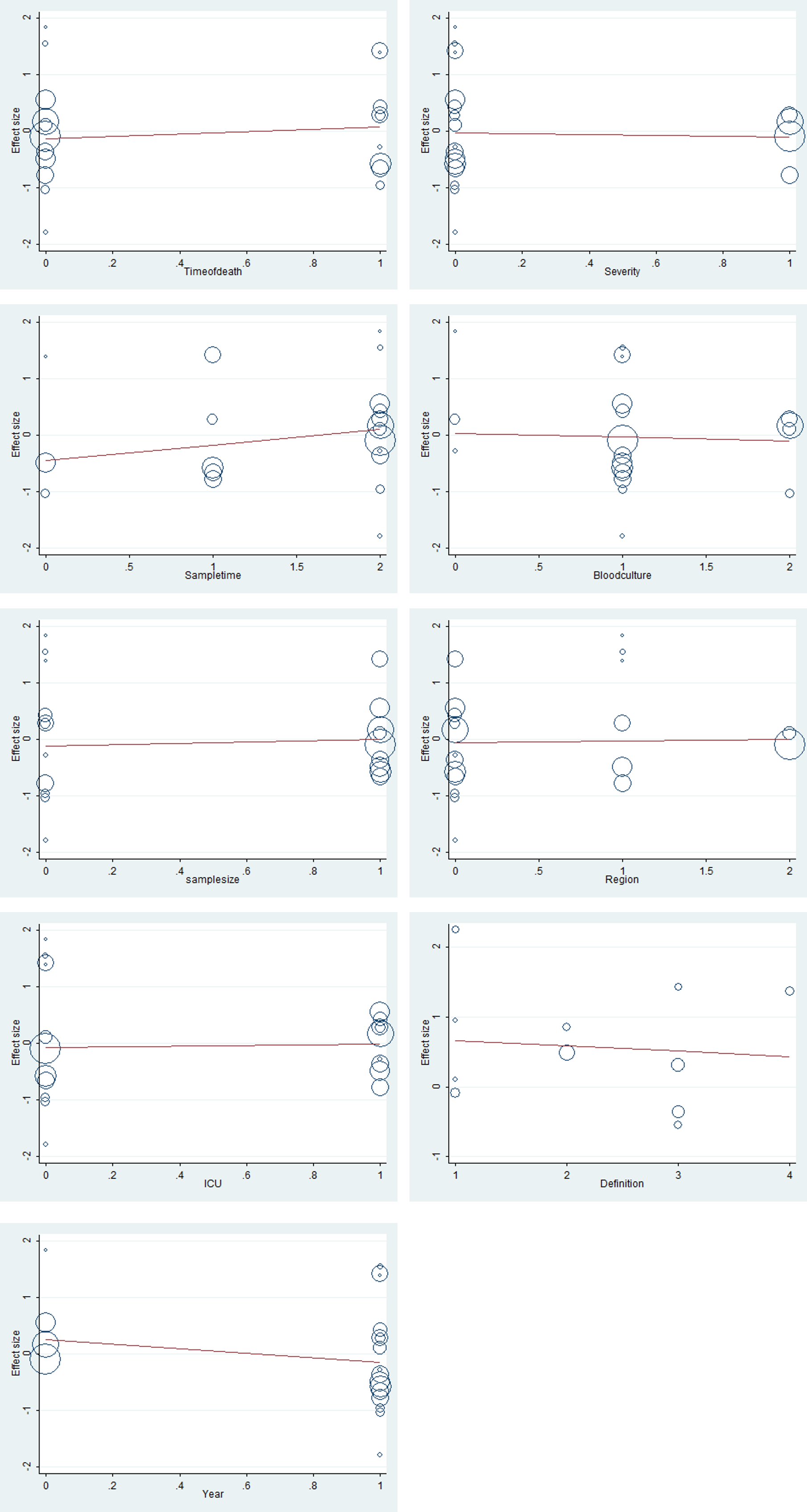


Survival


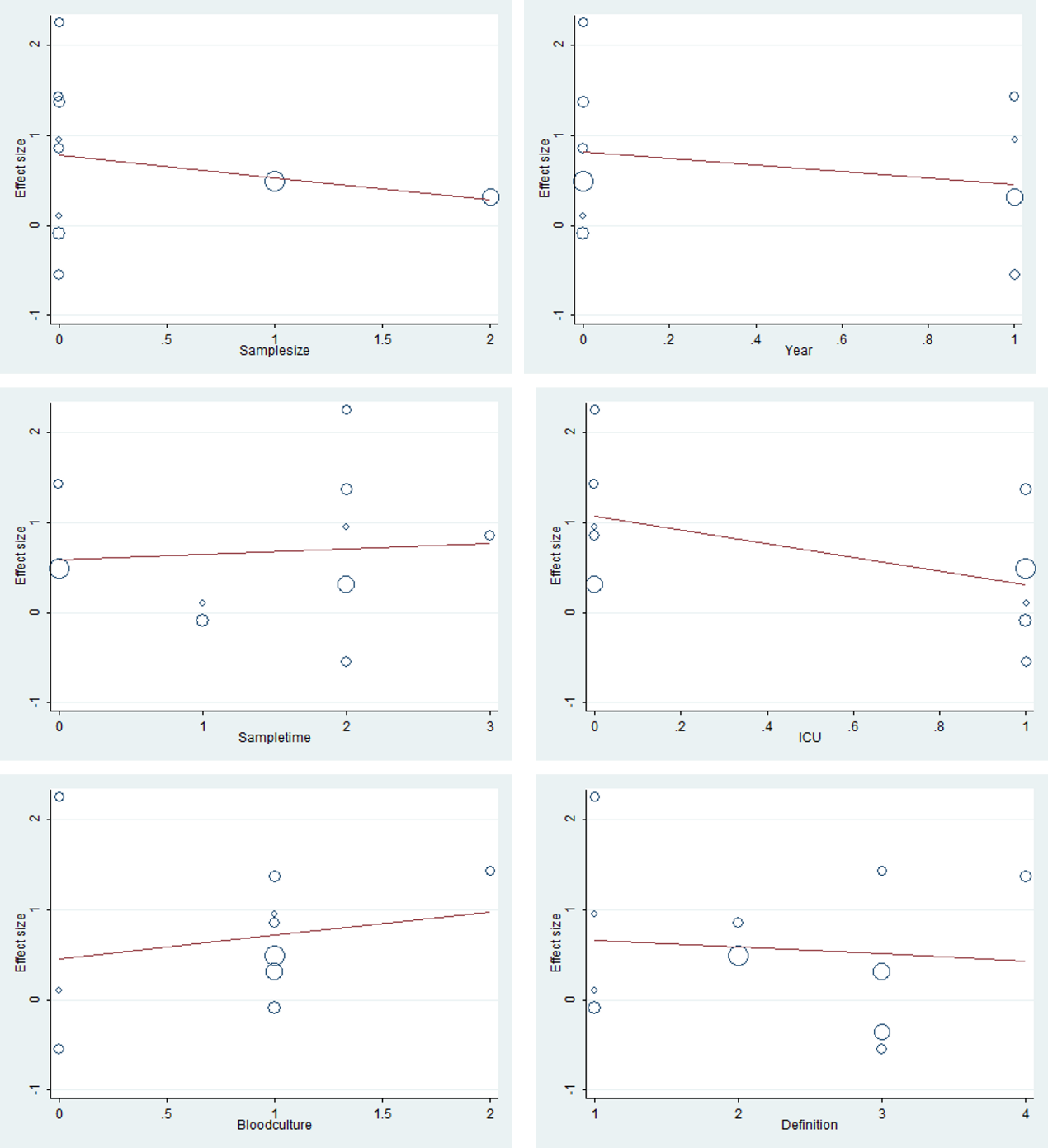


Sever sepsis


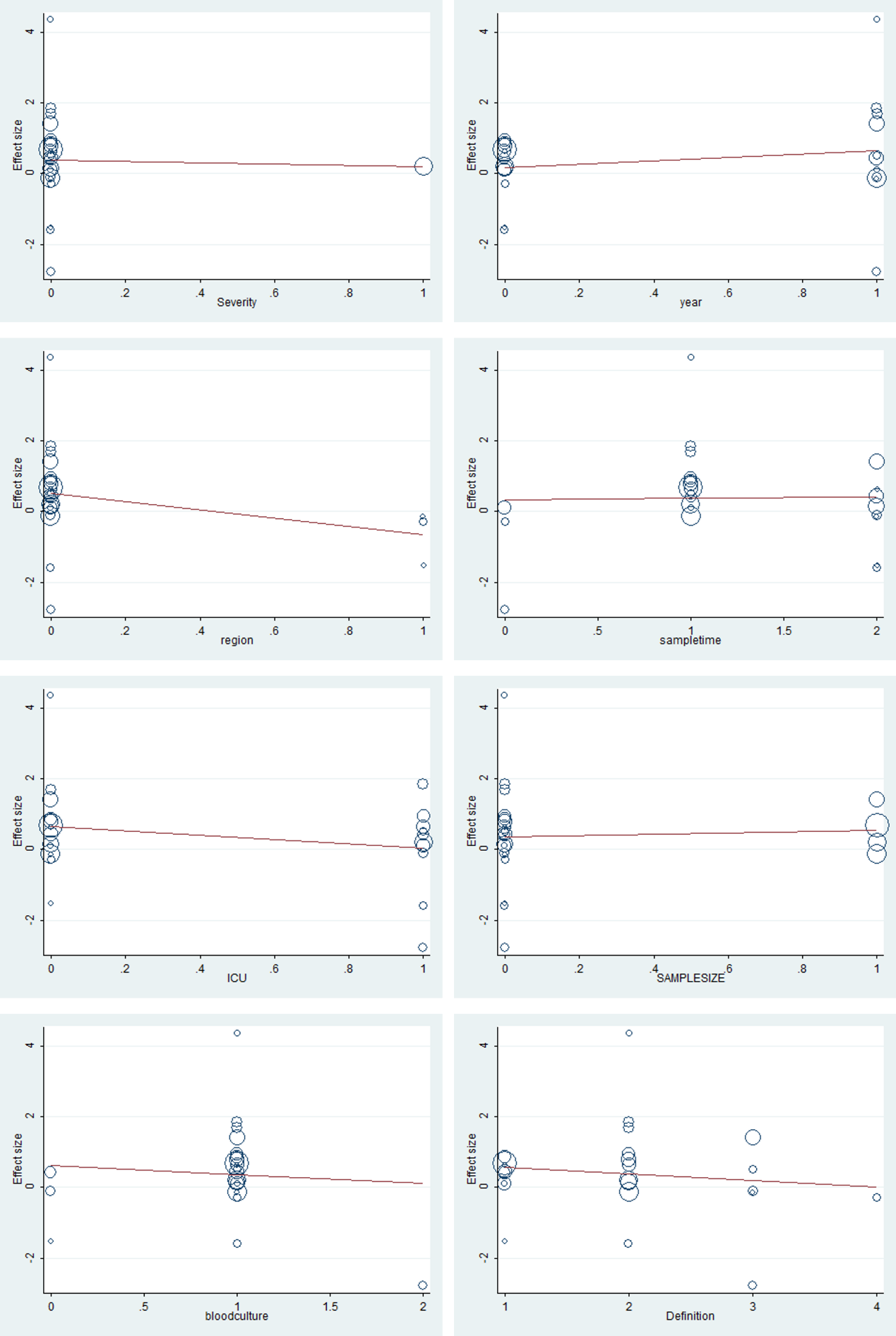


CRP


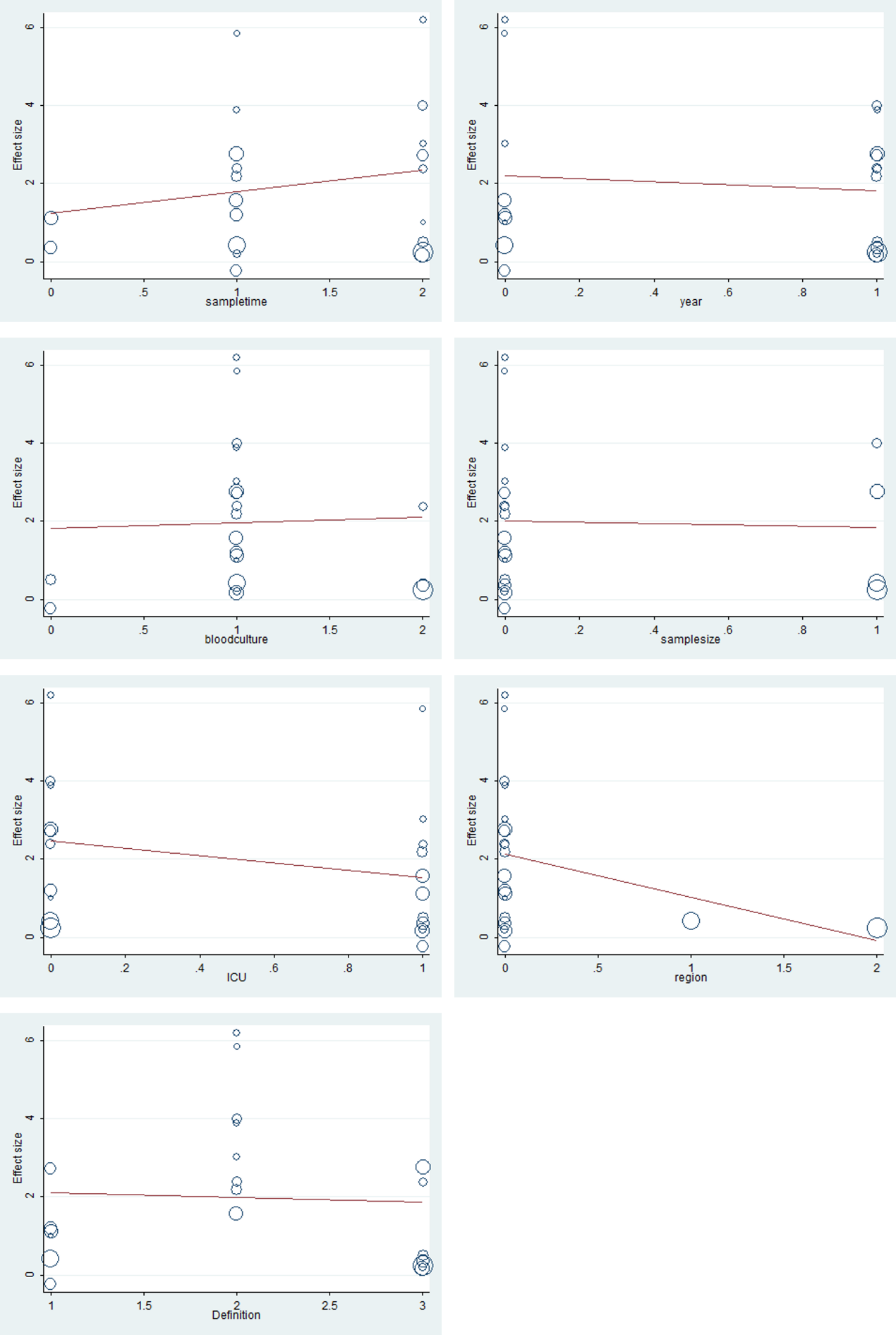


PCT


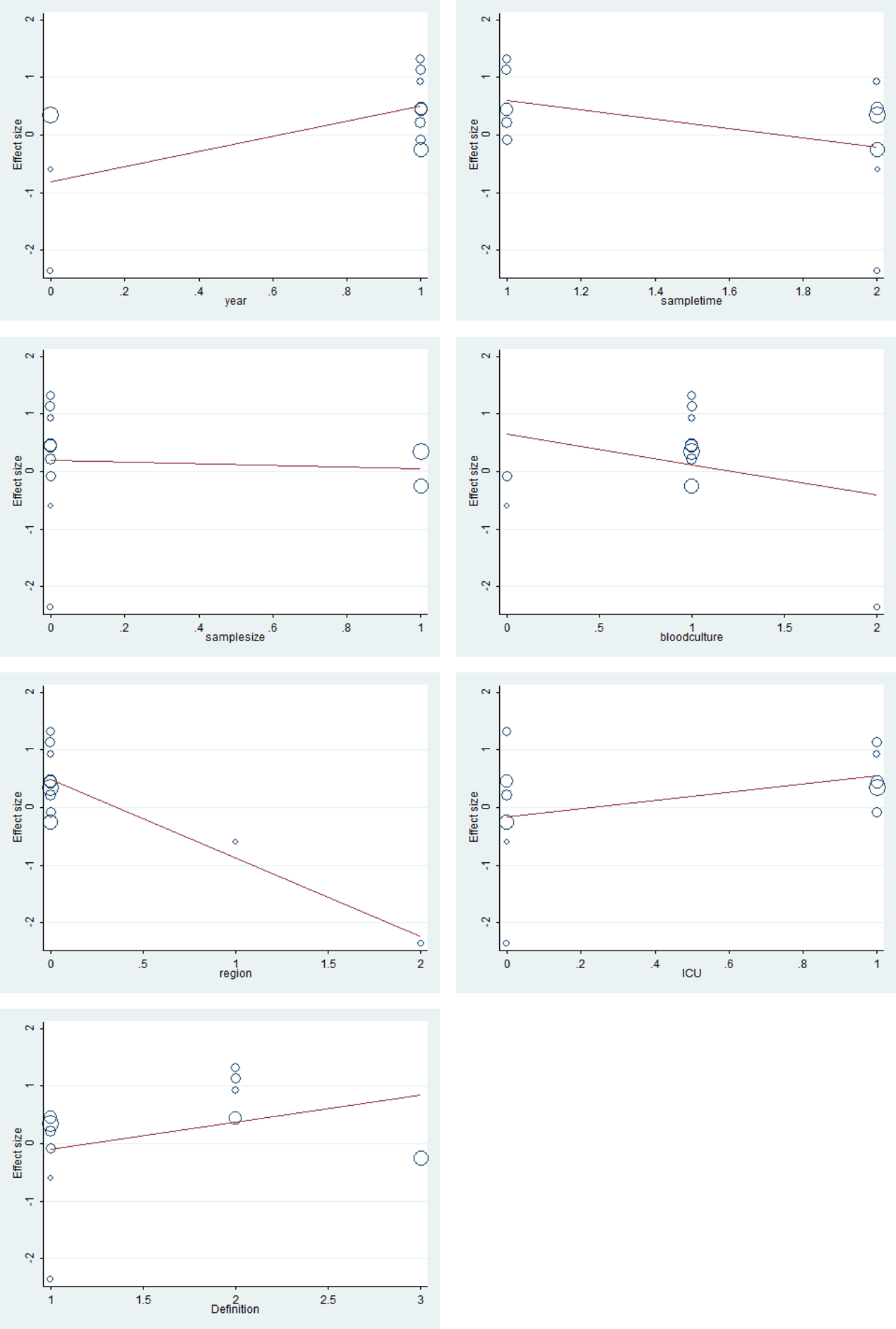


APACHE Ⅱ
