# Supplementary material for: Prognostic differences in sepsis caused by gram-negative bacteria and gram-positive bacteria: a systematic review and meta-analysis
Source: Crit Care. 2023 Nov 30;27:467. doi: 10.1186/s13054-023-04750-w (PMC10691150; doi:10.1186/s13054-023-04750-w)
Supplement: Supplementary file 6 — Additional file 6. Plot of funnel. [file 13054_2023_4750_MOESM6_ESM.docx]

Plot of funnel

- Survival
- Septic shock/Severe sepsis
- CRP
- PCT
- WBC
- APACHE Ⅱ
- SOFA


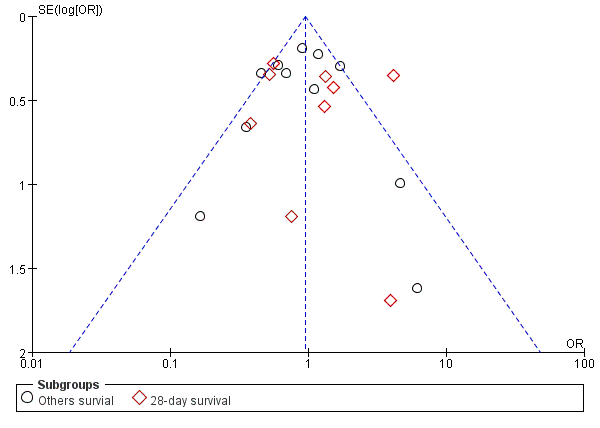


Survival


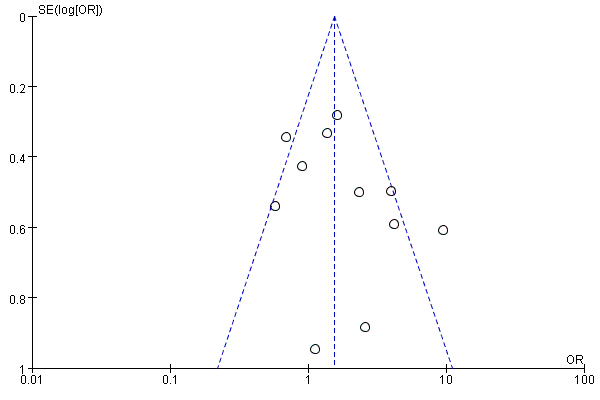


Septic shock/Severe sepsis


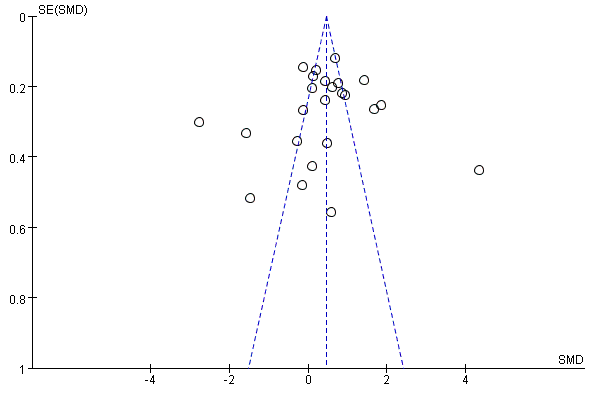


CRP


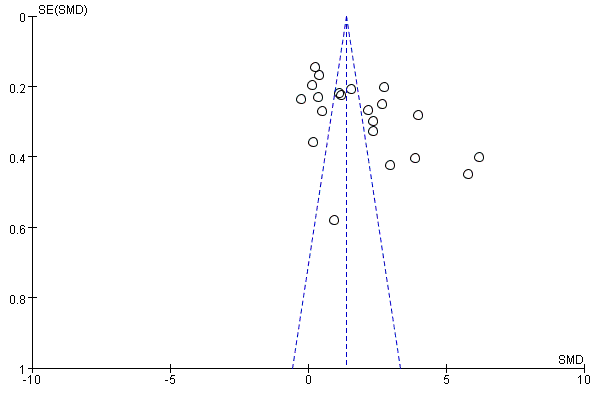


PCT


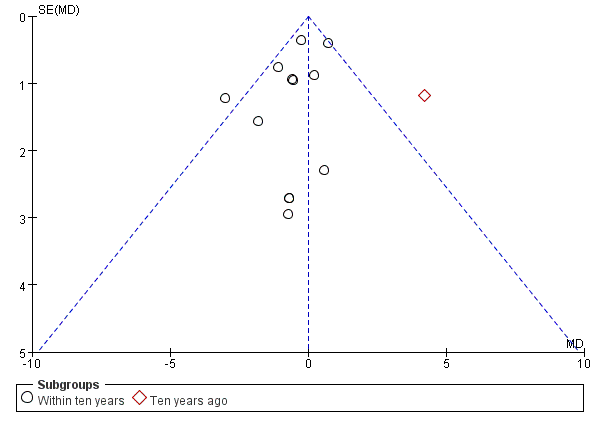


WBC


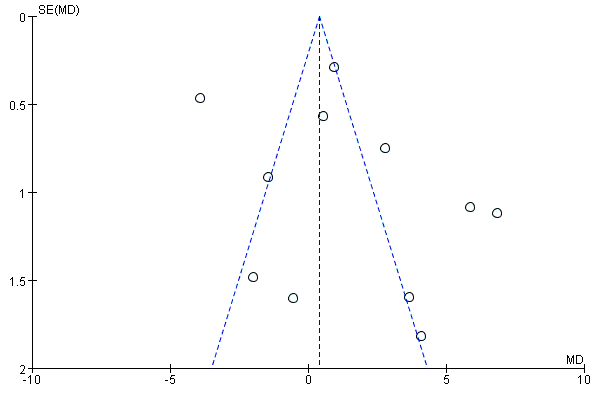


APACHE Ⅱ


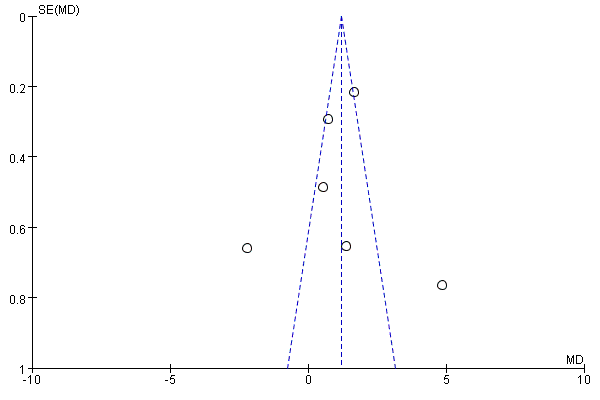


SOFA
