# Supplementary material for: Prognostic differences in sepsis caused by gram-negative bacteria and gram-positive bacteria: a systematic review and meta-analysis
Source: Crit Care. 2023 Nov 30;27:467. doi: 10.1186/s13054-023-04750-w (PMC10691150; doi:10.1186/s13054-023-04750-w)
Supplement: Supplementary file 7 — Additional file 7. Forest plots. [file 13054_2023_4750_MOESM7_ESM.docx]

**Forest plots**

- Forest plots of survival (subgroup analysis according to bacteremia)
- Forest plots of survival (subgroup analysis according to the definition of sepsis)
- Forest plots of TNF-α and IL-6
- Forest plots of coagulation function

**
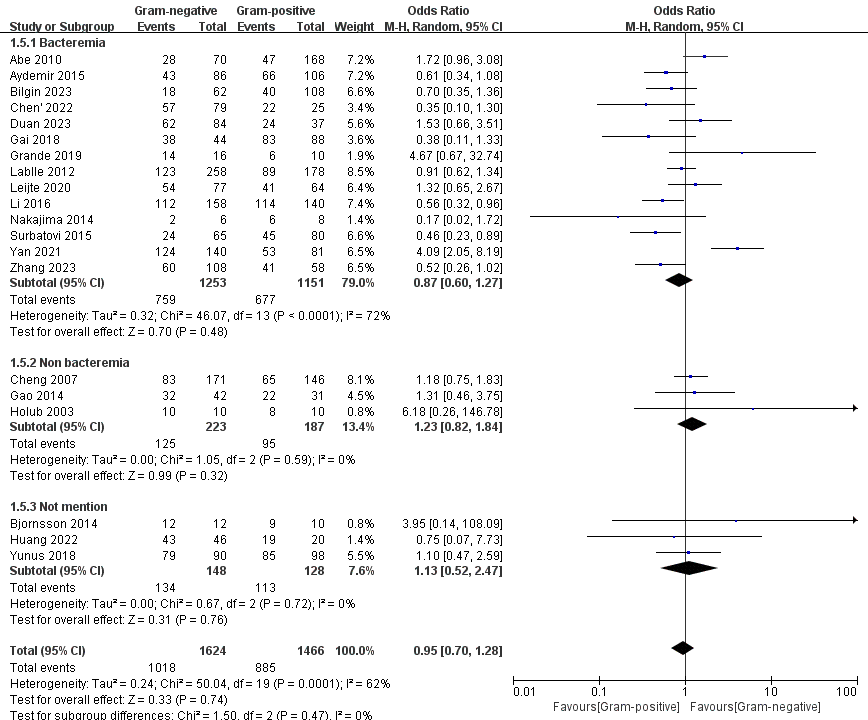
**

Forest plots of survival (subgroup analysis according to bacteremia)


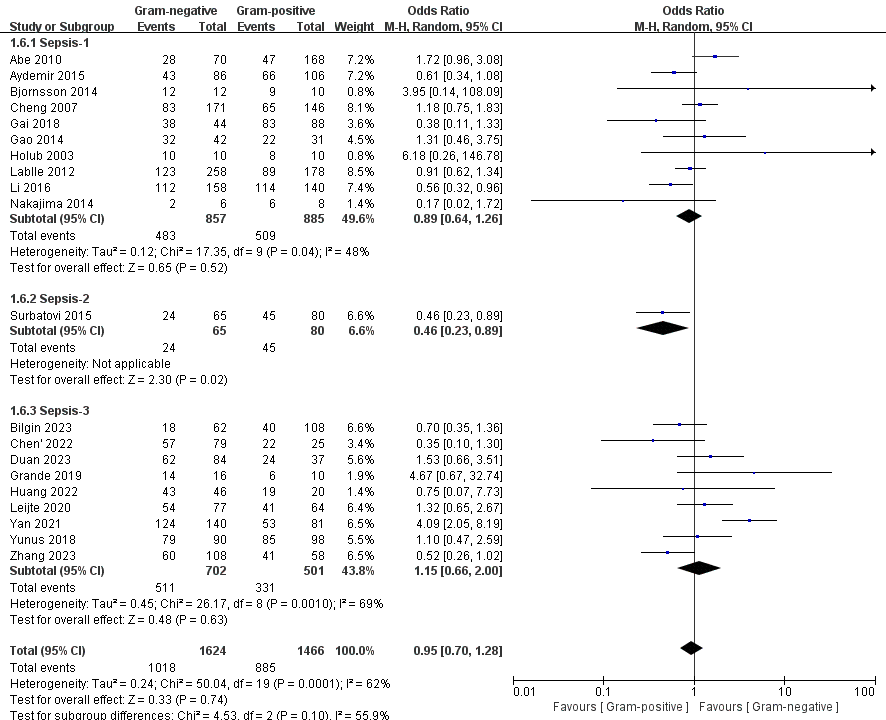


Forest plots of survival (subgroup analysis according to the definition of sepsis)

**
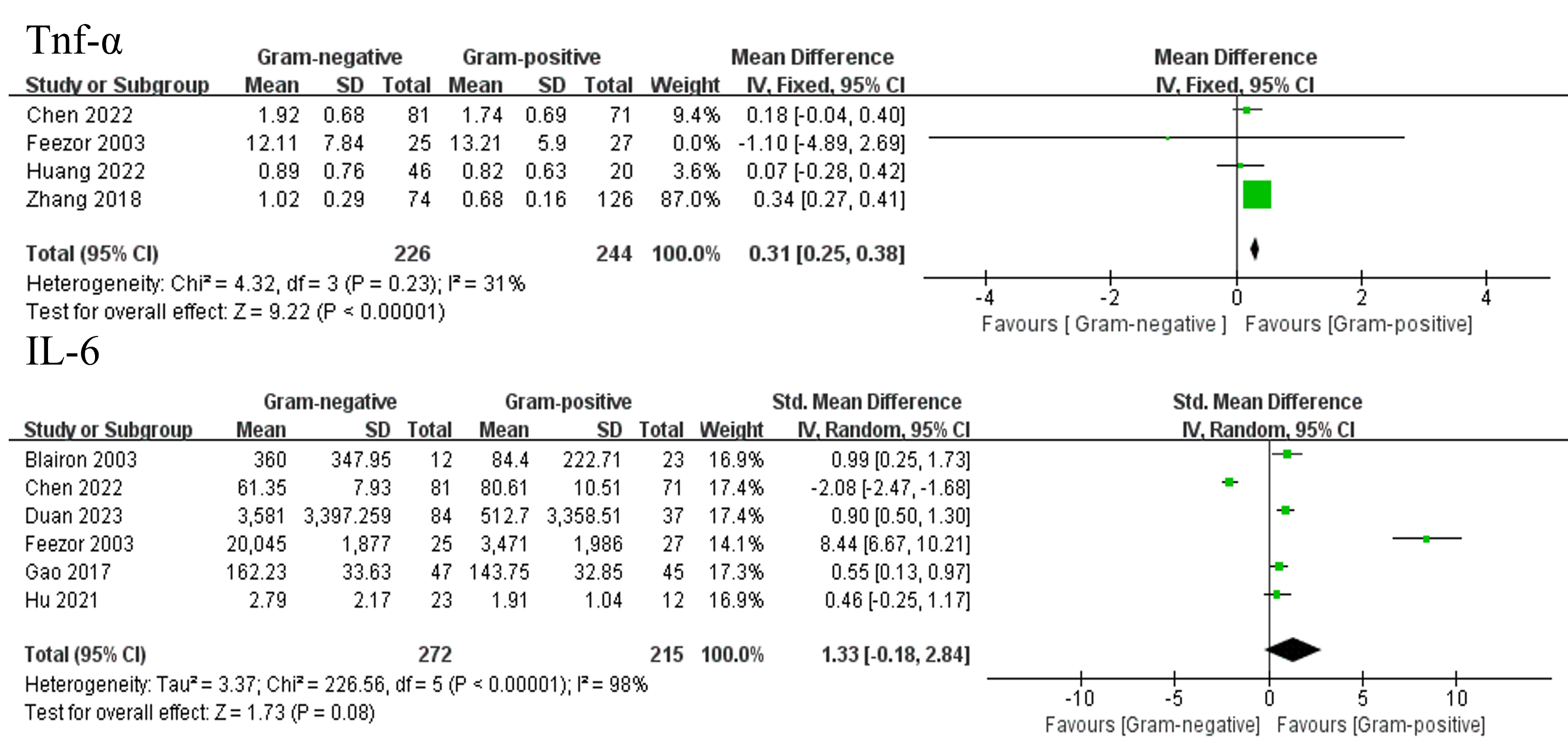
**

Forest plots of TNF-α and IL-6


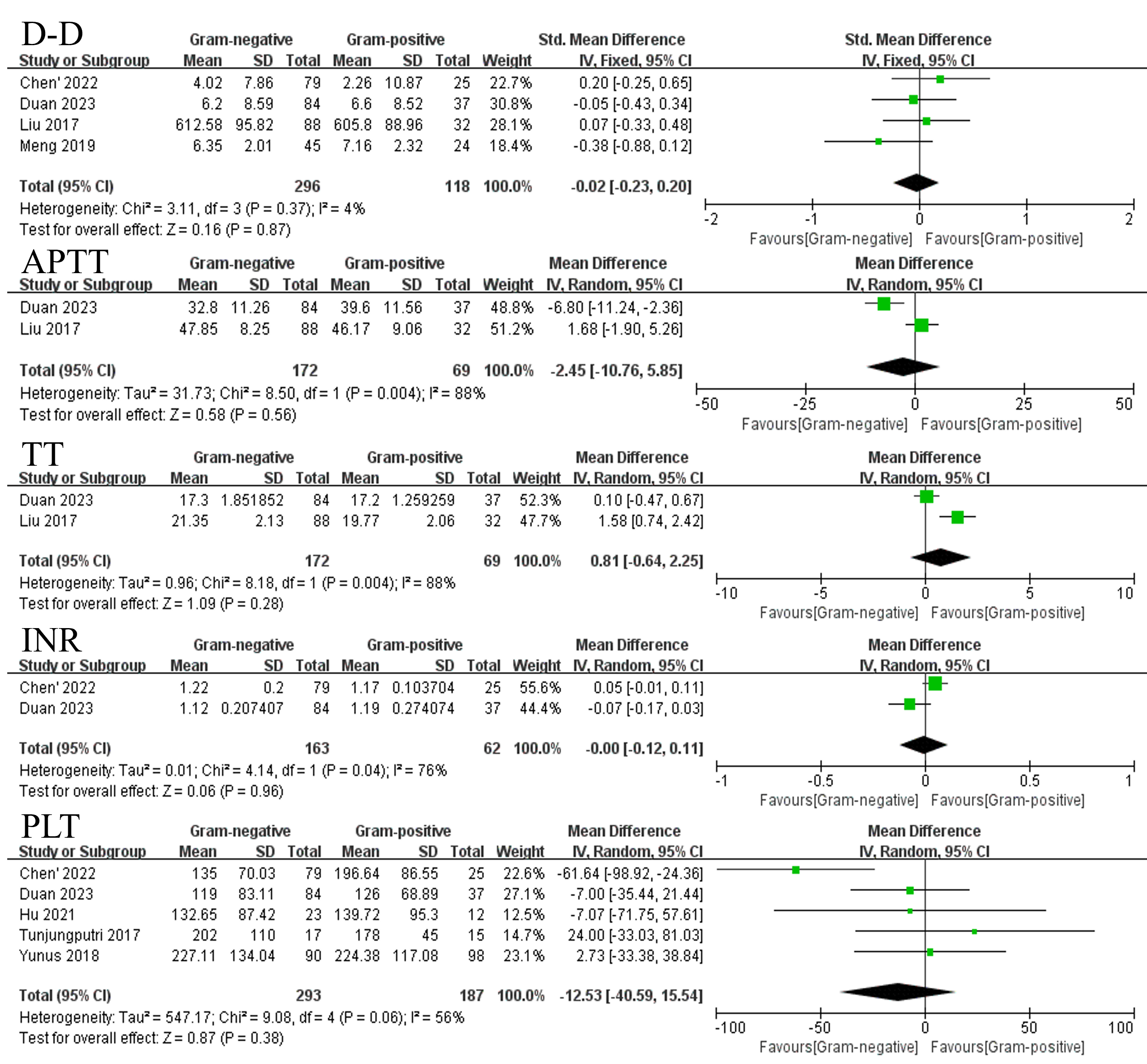


Forest plots of coagulation function
